# Adhesion G Protein-Coupled Receptor G2 Promotes Hepatocellular Carcinoma Progression and Serves as a Neutrophil-Related Prognostic Biomarker

**DOI:** 10.3390/ijms242316986

**Published:** 2023-11-30

**Authors:** Qian Wu, Pei Wang, Qihang Peng, Zhongcui Kang, Yiting Deng, Jiayi Li, Ying Chen, Jin Li, Feng Ge

**Affiliations:** 1 College of Life Science, Yangtze University, Jingzhou 434025, China; 2 State Key Laboratory of Freshwater Ecology and Biotechnology, Institute of Hydrobiology, Chinese Academy of Sciences, Wuhan 430072, China; gefeng@ihb.ac.cn

**Keywords:** *ADGRG2*, hepatocellular carcinoma, prognostic marker, neutrophil, miR-326, immune inflammation

## Abstract

Adhesion G protein-coupled receptor G2 (ADGRG2) is an orphan adhesion G protein-coupled receptor (GPCR), which performs a tumor-promoting role in certain cancers; however, it has not been systematically investigated in hepatocellular carcinoma (HCC). In the current study, we utilized multiple databases to analyze the expression and diagnostic and prognostic value of ADGRG2 in HCC and its correlation with immune infiltration and inflammatory factors. The function and upstream regulatory miRNA of ADGRG2 were validated through qPCR, Western blot, CCK8, wound healing, and dual luciferase assays. It turned out that ADGRG2 was significantly higher in HCC and had a poor survival rate, especially in AFP ≤ 400 ng/mL subgroups. Functional enrichment analysis suggested that ADGRG2 may be involved in cancer pathways and immune-related pathways. In vitro, siRNA-mediated ADGRG2 silencing could inhibit the proliferation and migration of Huh7 and HepG2 cells. There was a highly significant positive correlation between ADGRG2 and neutrophils. Moreover, NET-related genes were filtered and confirmed, such as ENO1 and S100A9. Meanwhile, the high expression of ADGRG2 was also accompanied by the highest number of inflammatory cytokines, chemokines, and chemokine receptors and good immunotherapy efficacy. Finally, AGDGR2 may be sensitive to two drugs (PIK-93 and NPK76-II-72-1) and can be targeted by miR-326. In conclusion, ADGRG2 may serve as a novel biomarker and drug target for HCC diagnosis, immunotherapy, and prognosis and was related to neutrophils and the inflammatory process of liver cancer development.

## 1. Introduction

Hepatocellular carcinoma (HCC) is one of the most common cancers, with a high mortality rate worldwide [1,2]. In 2018, there were over 782,000 HCC deaths and 841,000 new cases around the world [3]. Alcohol abuse, overweight, nonalcoholic fatty liver disease (NAFLD), and chronic hepatitis B or C virus infection represent some of the risk factors for HCC [4,5]. Most patients with early-stage HCC do not have obvious pathological symptoms, resulting in poor success and survival rates of radical surgery in late-stage patients and high drug resistance [6,7]. Some new treatments have been developed in the past decade, including molecular targeted therapy and immunotherapy [8]. Sorafenib, lenvatinib, apatinib, etc., which are inhibitors against vascular endothelial growth factor (VEGF) receptors, are among the first-line treatments for the targeted therapy of HCC. Immunotherapy is the most recent therapeutic option for HCC, such as nivolumab and pembrolizumab, which are used to treat advanced HCC patients by preventing the immune checkpoint inhibitor PD-1 from interacting with its ligands, PD-L1; T lymphocytes are subsequently activated to achieve antitumor effects [9]. Both types of therapies mentioned above may result in some patients not responding or experiencing toxic reactions [10,11]. This might be connected to the complexity of tumor occurrence and progression and to tumor heterogeneity caused by the inflammatory and immune microenvironment in which liver cancer cells are located [12,13]. Therefore, developing novel diagnostic indicators and targeted therapies for HCC and gaining a deeper exploration of the molecular mechanism underlying the emergence and progression of HCC remains a challenging task.

Adhesion G protein-coupled receptor G2 (ADGRG2, also known as GPR64 or HE6) belongs to the family of G protein-coupled receptors (GPCRs), which are mostly expressed in epididymis and play an important role in male fertility [14,15]. It has been shown that ADGRG2 was highly elevated in some solid tumors and can facilitate cell growth and invasion of various cancer cells, such as Ewing sarcoma, parathyroid tumor, hormone cell adenoma, etc. [16,17,18]. Conversely, ADGRG2 was underexpressed in endometrial carcinoma and may be tumor-suppressing [19]. In addition, ADGRG2 has been implicated as an immune-related gene with independent prognostic significance for Ewing sarcoma, and it has the potential for drug delivery in antibody-based sarcoma therapy [20,21]. Treatment of HCC cells with modulated electro-hyperthermia (mEHT)-induced apoptosis led to a decreased mRNA expression of ADGRG2, suggesting that ADGRG2 may act as a cancer-causing gene in HCC [22]. At present, more than 30% of the global small molecule drug market targets GPCRs [23]. Their characteristics represent effective targets for molecular imaging and therapy, with broad potential value and development prospects [24]. However, the systematic analysis of the expression, prognosis, and treatment of ADGRG2 in cancers still remains unexplored.

In this study, we used a combination of bioinformatics and experimental validation methods to systematically evaluate the expression of ADGRG2 in HCC, as well as its correlation with diagnosis, prognosis, cancer occurrence and development, immune infiltration, and inflammation. In addition, we predicted the response of ADGRG2 expression to immunotherapeutic treatment in HCC and screened targeted drugs and miRNAs. These findings provided the theoretical basis for revealing ADGRG2 as an effective biomarker for HCC and a new therapeutic target.

## 2. Results

### 2.1. High Expression of ADGRG2 in HCC

**Figure 1:**
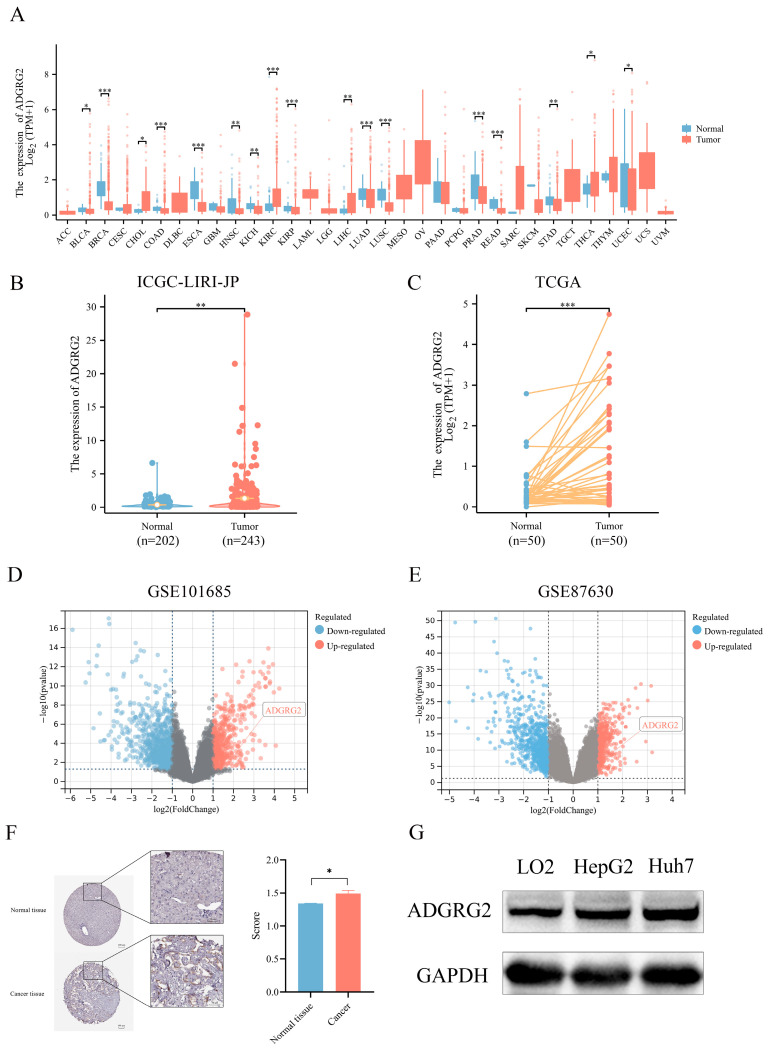
*ADGRG2* expression in pan-cancer and HCC. (**A**) The mRNA expression level of *ADGRG2* in 33 various malignant tumor types from TCGA database. (**B**) The expression of *ADGRG2* from the ICGC-LIRI-JP. (**C**) *ADGRG2* expression in 50 paired tumors along with normal tissues of HCC patients in TCGA. (**D**,**E**) *ADGRG2* expression was up-regulated in HCC samples from GSE101685 and GSE87630. (**F**) ADGRG2 protein levels in HCC and adjacent normal tissues were determined using the HPA database. (**G**) ADGRG2 protein levels were detected in LO2, HepG2, and Huh7 cell lines using Western blot. * *p* < 0.05, ** *p* < 0.01, *** *p* < 0.001.

We examined the expression of ADGRG2 in 33 kinds of cancers using TCGA (tumor and normal data) cohorts (Figure 1A), resulting in most cancer patients’ ADGRG2 being down-regulated, while it was preeminently higher in four types of cancer, including cholangiocarcinoma (CHOL), kidney renal clear cell carcinoma (KIRC), liver hepatocellular carcinoma (LIHC), and thyroid carcinoma (THCA). Then, the up-regulated expression of ADGRG2 was verified in unpaired HCC samples (202 normal tissues vs. 243 tumor tissues) in ICGC_LIHC (Figure 1B), paired HCC tissues (50 pairs) in TCGA (Figure 1C), and the GEO database (Figure 1D,E). Additionally, ADGRG2 protein expression was remarkably elevated in HCC samples, which was obtained from immunohistochemical results in the HPA database (Figure 1F). The same situation was also observed in cell lines (HCC cell lines HepG2 and Huh7 vs. normal liver cell line LO2) (Figure 1G).

### 2.2. High Expression of ADGRG2 Was Associated with Adverse Clinicopathological Factors and Worse Prognosis in HCC

**Figure 2:**
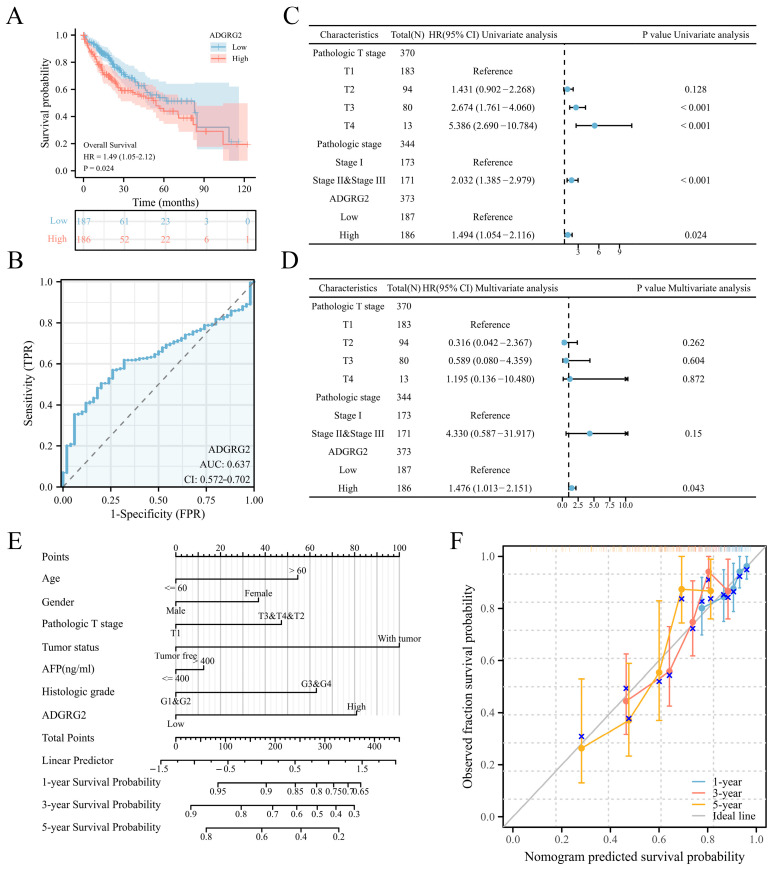
ADGRG2’s predictive significance in HCC patients. (**A**) OS curves between high- and low-ADGRG2 subgroups. (**B**) ROC curve of ADGRG2. (**C**,**D**) Univariate and multivariate Cox regression analysis of ADGRG2 expression for OS. (**E**,**F**) A nomogram and calibration curves for the calculation of 1-, 3-, and 5-year OS.

According to the expression levels of ADGRG2, 374 HCC patients in the TCGA (LIHC) dataset were separated into low- (*n* = 187) and high-level (*n* = 187) groups. ADGRG2 expression was found to be substantially associated with pathologic stage (*p* < 0.05), α Fetoprotein (AFP) (ng/mL) (*p* < 0.01), and an OS event (*p* < 0.05) (Table 1). Interestingly, patients with AFP (≤400 ng/mL) and higher levels of ADGRG2 expression were more ill than patients with other features and had low OS (*p* < 0.05) (Figure S1). Furthermore, the Kaplan–Meier analysis indicated that a high ADGRG2 expression was strongly connected with lower OS (*p* = 0.024) (Figure 2A), and the value of ROC demonstrated that ADGRG2 may serve as a possible diagnostic biological marker for HCC, considering an area under the curve (AUC) of 0.637 (Figure 2B). In order to determine whether ADGRG2 was a distinct prognostic marker for HCC patients, univariate as well as multivariate Cox regression analysis were performed. According to the findings of the univariate Cox regression analysis, pathologic T stage, pathologic stage, and ADGRG2 expression were closely related to OS (*p* < 0.05) (Figure 2C). Moreover, ADGRG2 expression was an independent predictor affecting HCC prognosis (HR = 1.476, *p* = 0.043) (Figure 2D) in the multivariate Cox regression approach. Subsequently, a nomogram was established on the basis of the results of the Cox analysis, which revealed that the C-index of the nomogram was 0.678 (95% confidence interval: 0.648–0.709), and the 1-, 3-, and 5-year calibration curves all exhibited a good capacity for predicting the prognosis of HCC (Figure 2E,F). Taken together, ADGRG2 expression can be used as an independent prognostic indicator for HCC patients.

### 2.3. Enrichment Analysis of ADGRG2-Related DEGs in HCC

**Figure 3:**
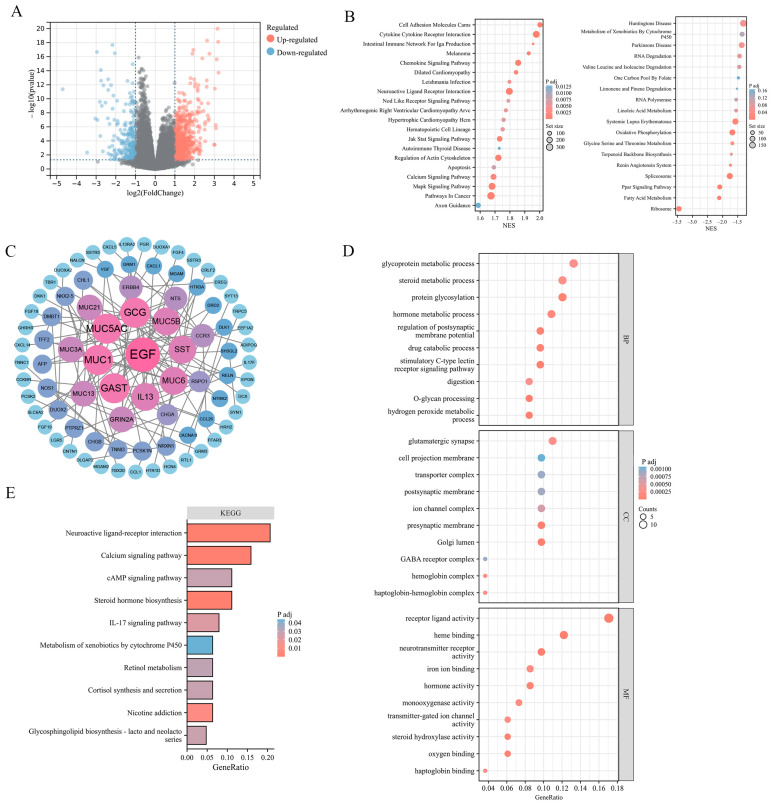
*ADGRG2*-related DEGs and functional enrichment analysis in HCC. (**A**) Volcano plot of *ADGRG2*-related DEGs. The markedly down-regulated and up-regulated DEGs are represented by blue and red dots, respectively. (**B**) Gene set enrichment analysis (GSEA) of DEGs. (**C**) PPI network for DEGs. (**D**,**E**) The GO and KEGG analysis based on the hub DEGs.

To explore the potential effect of ADGRG2 in HCC, 881 DEGs were identified, of which 615 were up-regulated and 266 down-regulated (Figure 3A). The findings of the gene set enrichment analyses (GSEA) indicated that ADGRG2-related DEGs were involved in the positive regulation of pathways in cancer (JAK-STAT, MAPK, and Calcium signaling pathway), cell adhesion molecules cams, cytokine–cytokine receptor interaction, and the negative regulation of oxidative phosphorylation (Figure 3B). Furthermore, 84 hub DEGs were filtered from the PPI network of ADGRG2-related DEGs via STRING (Figure 3C). The GO enrichment results suggested that these hub DEGs were predominantly linked with protein glycosylation, stimulatory C-type lectin receptor signaling pathway, Golgi lumen, and iron ion binding (Figure 3D). The KEGG pathway analysis indicated that the enrichment terms of these hub DEGs mainly participated in the cAMP signaling pathway, IL-17 signaling pathway, and Calcium signaling pathway (Figure 3E). These findings indicated that the ADGRG2 may be a tumorigenesis- and immune-related factor in HCC.

### 2.4. Knockdown of ADGRG2 Inhibited the Proliferation and Migration of HCC Cells

**Figure 4:**
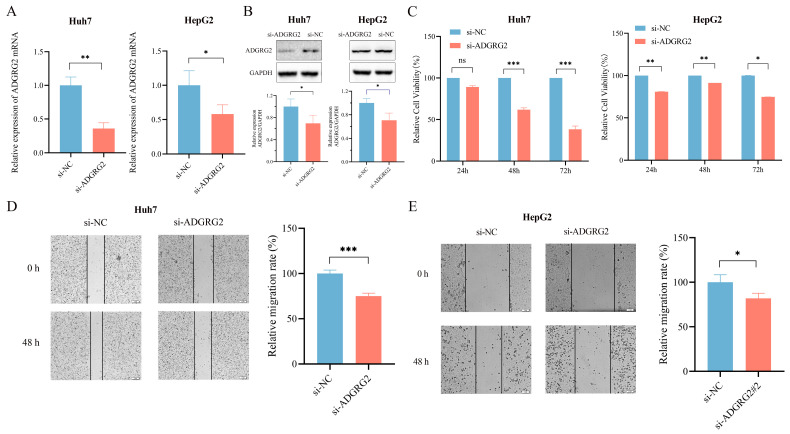
Knockdown of ADGRG2 inhibited HepG2 and Huh7 cells’ migration and proliferation. (**A**,**B**) Verification of ADGRG2 expression changes after transfection of si-NC and si-ADGRG2 at the mRNA and protein levels, respectively. (**C**) The cell proliferation capacity and (**D**,**E**) migration of Huh7 and HepG2 after silencing of ADGRG2 by CCK-8 and wound healing assays. * *p* < 0.05, ** *p* < 0.01, *** *p* < 0.001, ns: no significance.

To further determine the possible biological function of ADGRG2 in HCC, we transfected ADGRG2-specific siRNAs into Huh7 and HepG2 cells and found that ADGRG2 was successfully down-regulated in mRNA and protein levels, respectively (Figure 4A,B). CCK-8 and the wound healing assays showed that the growth and migration of Huh7 and HepG2 cells were inhibited after silencing of ADGRG2 (Figure 4C–E).

### 2.5. Associations between ADGRG2 and Neutrophil Infiltration in HCC

**Figure 5:**
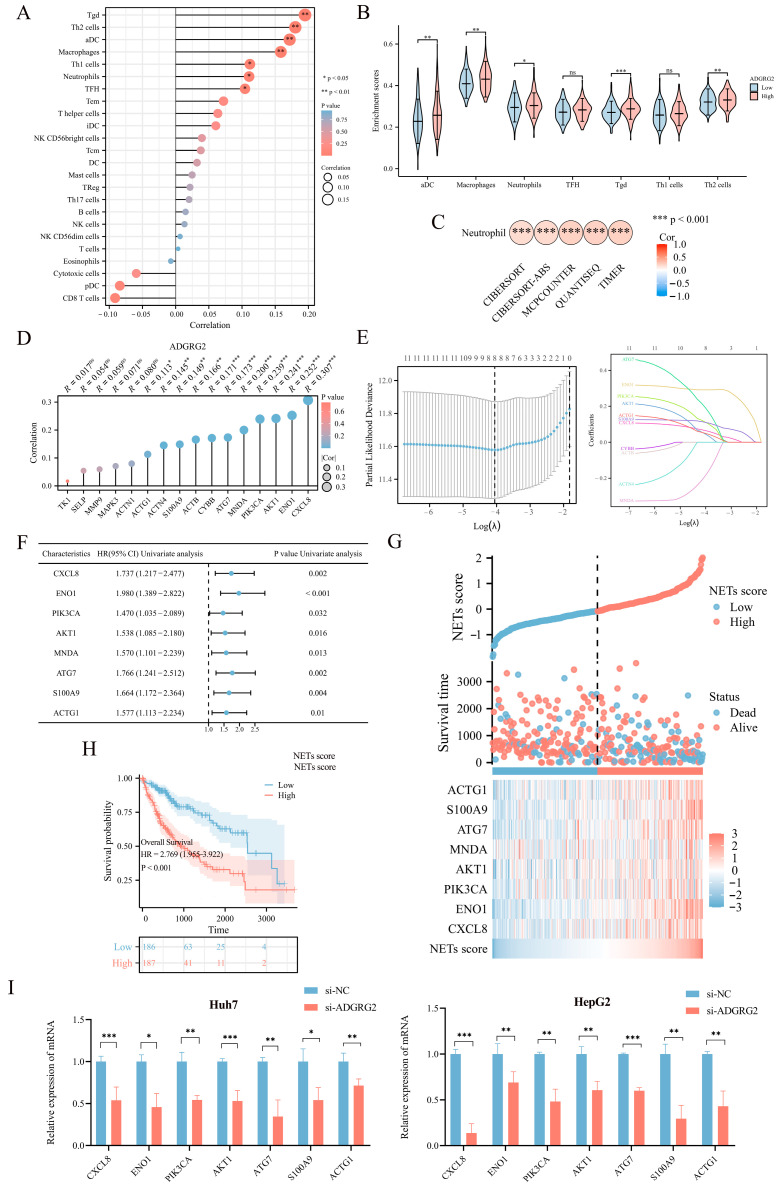
Correlation analysis between ADGRG2 expression and immune infiltration. (**A**) Spearman’s correlations of ADGRG2 expression with the infiltration levels of various immune cells in HCC tissues. (**B**) Score of immune cell enrichment between groups with high and low ADGRG2. (**C**) Analysis of the correlation between ADGRG2 and neutrophils via five algorithms. (**D**) The correlation between ADGRG2 and sixteen prognosis-related NET-related genes. (**E**) Screening for NET-related genes closely associated with prognosis using LASSO regression analysis. (**F**) Univariate Cox regression analysis of eight NET-associated genes. (**G**) Survival time and NET score distribution for each patient in the TCGA_LIHC cohort. (**H**) Kaplan–Meier survival curve of the high or low NETs score. (**I**) The mRNA level of seven NET-related genes in HepG2 and Huh7 after silencing of AGDGR2. * *p* < 0.05, ** *p* < 0.01, *** *p* < 0.001, ns: no significance.

As ADGRG2-associated genes were strongly enriched in immune-related signaling pathways, we further explore the connection between ADGRG2 and tumor immunity. The ssGSEA calculation results indicated that ADGRG2 expression had a significantly positive correlation with aDC (r = 0.172, *p* < 0.001), macrophages (r = 0.158, *p* = 0.002), neutrophils (r = 0.111, *p* = 0.032), and multiple types of T cells, including Tgd (r = 0.195, *p* < 0.001), Th2 cells (r = 0.180, *p* < 0.001), Th1 cells (r = 0.112, *p* = 0.031), and TFH (r = 0.104, *p* = 0.043) (Figure 5A). Tgd, Th2 cells, aDC, macrophages, and neutrophils were markedly concentrated in the ADGRG2 high-expression group (Figure 5B). Notably, ADGRG2 was dramatically correlated with neutrophils according to five algorithms in HCC (Figure 5C), and most markers in neutrophils were connected with tumor-associated neutrophils (TAN) and inflammation (Table 2).

Neutrophils release neutrophil extracellular traps (NETs) in conditions such as inflammation and cancer, which contribute to cancer progression [25]. To identify NET-related signatures in HCC, we first screened sixteen NET-related genes with prognostic potential (Table S1), among which eleven genes showed a significant positive correlation with ADGRG2 (Figure 5D). Then, the eleven NET-related genes were submitted to LASSO regression analysis to obtain eight nonzero coefficients of the NET-related genes (Figure 5E). Next, Cox regression analysis was conducted on the eight NET-related genes, and univariate Cox analysis showed that all these eight genes (CXCL8, ENO1, PIK3CA, AKT1, MNDA, ATG7, S100A9, and ACTG1) have HR > 1 (*p* < 0.05) (Figure 5F). Subsequently, NET scores were produced based on their standardized levels, and TCGA-LIHC patients were separated into two groups based on their NET score: high-NETs and low-NETs (Figure 5G). Finally, the relationship between the NET score and prognosis was analyzed using a Kaplan–Meier curve, which showed that patients with HCC with a higher NET score had a worse prognosis (*p* < 0.001) (Figure 5H). Furthermore, we identified that the mRNA level of seven NET-related genes (CXCL8, ENO1, PIK3CA, AKT1, ATG7, S100A9, and ACTG1) were down-regulated in HepG2 and Huh7 cells after silencing of AGDGR2 (Figure 5I). These results suggested that ADGRG2 may recruit neutrophils and participate in the release of NETs.

### 2.6. The Potential Role of ADGRG2 in Inflammation

**Figure 6:**
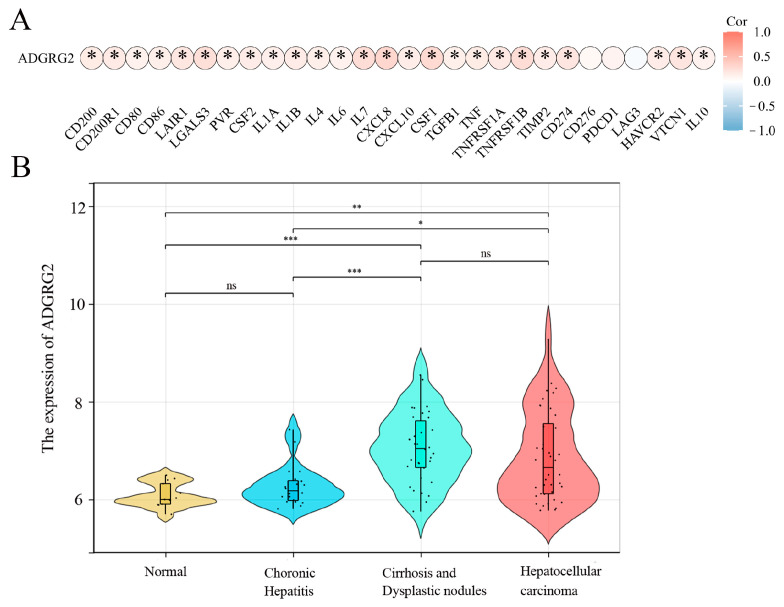
ADGRG2 was associated with the progression of inflammation in HCC. (**A**) The association of ADGRG2 expression with inflammatory cytokines in HCC. (**B**) The expression status of ADGRG2 at different stages of HCC tumorigenesis according to GSE89377. * *p* < 0.05, ** *p* < 0.01, *** *p* < 0.001, ns: no significance.

A growing amount of evidence suggests that uncontrolled or excessive production of NETs is associated with the exacerbation of inflammation and cancer metastasis, and inflammation is a key driver in the pathogenesis of hepatocellular carcinoma [26]. We wondered whether ADGRG2 participated in inflammation as part of liver diseases. The analysis of the correlation between inflammatory cytokines and ADGRG2 showed that ADGRG2 had a positive correlation with most cytokines, including the classic liver inflammatory molecules: IL-6, IL-1α, IL-1β, TNF, and IL-10 (Figure 6A). Furthermore, the GSE89377 dataset was used to evaluate the expression status of ADGRG2 in HCC tumorigenesis. As shown in Figure 6B, the ADGRG2 expression was more elevated in liver cirrhosis and hepatocellular carcinoma than in the normal and chronic hepatitis groups, which may imply that ADGRG2 was tied to inflammation and the occurrence of HCC.

Chemokines can induce cell migration and the development of inflammation [27], and some chemokine receptors have been demonstrated to have seven transmembrane structures and being coupled to G proteins with multiple conserved motifs [28]. Hence, it was of interest to explore the potential role of ADGRG2 with chemokines and their receptors in HCC. The correlation of the ADGRG2 expression level with chemokines and most chemokine receptors was significant and positive, for instance, with CCL1, CCL2, CCL11, CCL20, CCL22, CCL26, CCL28, and CXC chemokines (Tables S2 and S3). This meant that ADGRG2 may be involved in the progression of HCC through these chemokines.

### 2.7. Correlation of ADGRG2 Expression with Immunotherapy and Drug Sensitivity

**Figure 7:**
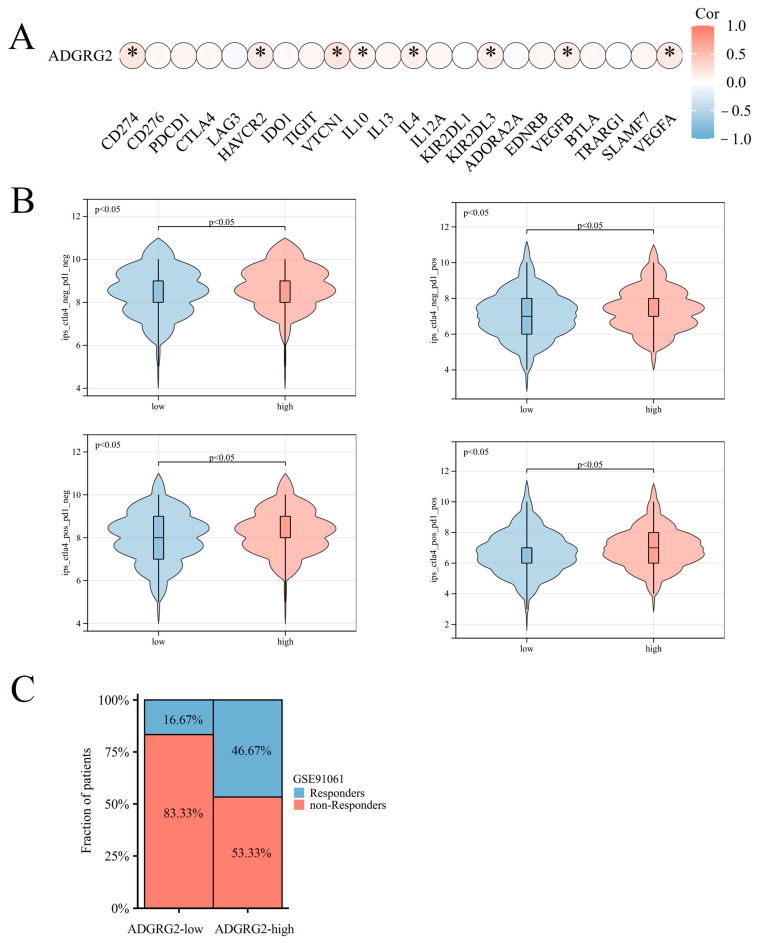
The relationship between ADGRG2 and immune checkpoint inhibitors and immunotherapy response. (**A**) Correlation analysis between ADGRG2 expression and immune checkpoint inhibitors in HCC. (**B**) The difference in IPS between the ADGRG2 high-expression group and ADGRG2 low-expression group. (**C**) Response of the high- and low-ADGRG2 subgroups in the GSE91061 cohort. * *p* < 0.05.

Considering that ADGRG2 was closely related to immunity and inflammation, we further analyzed its role in immunotherapy. However, ADGRG2 had a substantial positive correlation with only a small number of inhibitory immune checkpoints, such as CD274, HAVCR2, VTCN1, IL-10, IL-4, KIR2DL3, VEGFB, and VEGFA (Figure 7A), and ADGRG2 was associated with more immune checkpoints in pathologic stage T2 and T3 and T4 (vs. T1), AFP ≤ 400 subgroup (vs. >400), and mild and severe adjacent hepatic tissue inflammation (vs. none) (Figure S2). Immune checkpoint blockers (ICBs) are currently the most promising cancer treatments. To investigate the ability of ADGRG2 to respond to immunotherapy, we explored the relationship between ADGRG2 expression and immunophenoscore (IPS) in different groups. Figure 7B showed that the ADGRG2 high-expression group all exhibited a higher IPS than the low-expression group. Meanwhile, the ADGRG2 high-expression group had more responders than the ADGRG2 low-expression group in the GSE91061 cohort after receiving anti-PD-1 therapy (Figure 7C), which was consistent with the previous results. This suggested that patients with high ADGRG2 expression may respond better to immunotherapy.

**Figure 8:**
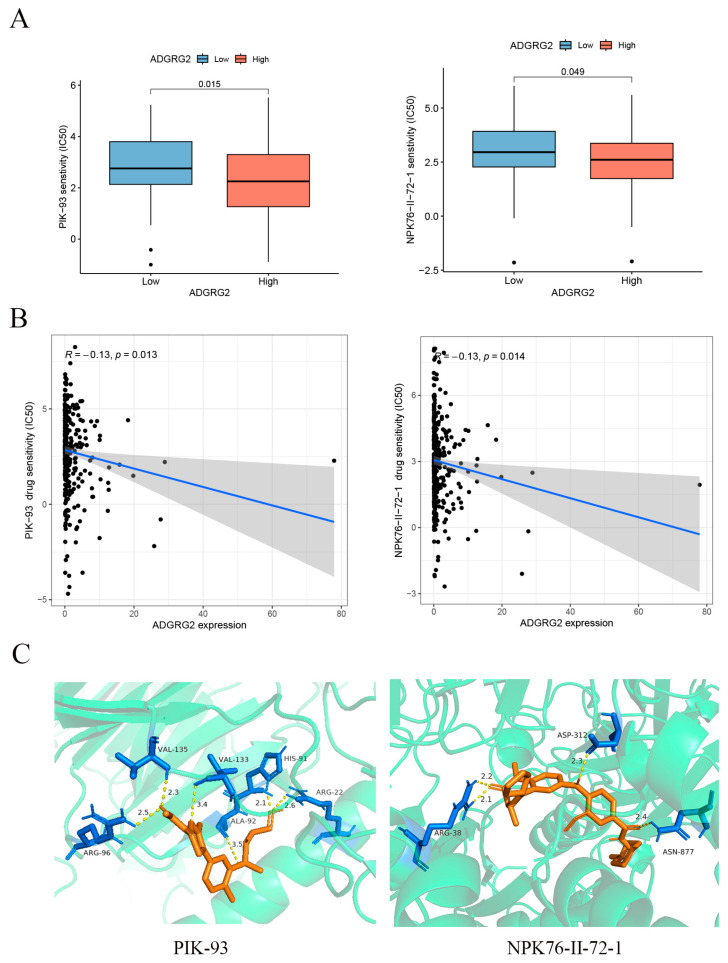
Correlation between ADGRG2 and drug sensitivity. (**A**) IC50 of drugs in different groups of ADGRG2. (**B**) The relationship between ADGRG2 and drug sensitivity. The black dots depict the expression value of ADGRG2 in each sample, corresponding to the drug’s IC50 value. (**C**) Molecular docking results of PIK-93 and NPK76-II-72-1 to ADGRG2.

GPCRs are the most important family of cell signal receptors, which are usually used for targeted drug development [23]. We attempted to predict drugs that can effectively target ADGRG2. The relationship between anticancer drugs and ADGRG2 showed that the IC50s of PIK-93 and NPK76-II-72-1 were lower in the ADGRG2 high-expression group (Figure 8A), and ADGRG2 was significantly negatively correlated with the IC50s of PIK-93 and NPK76-II-72-1 (Figure 8B). More importantly, the two drugs had a strong affinity with ADGRG2, calculated via molecular docking (PIK-93: −6.3 kcal/mol, NPK76-II-21-1: −8.8 kcal/mol) (Figure 8C).

### 2.8. Prediction of miRNAs Targeting ADGRG2

**Figure 9:**
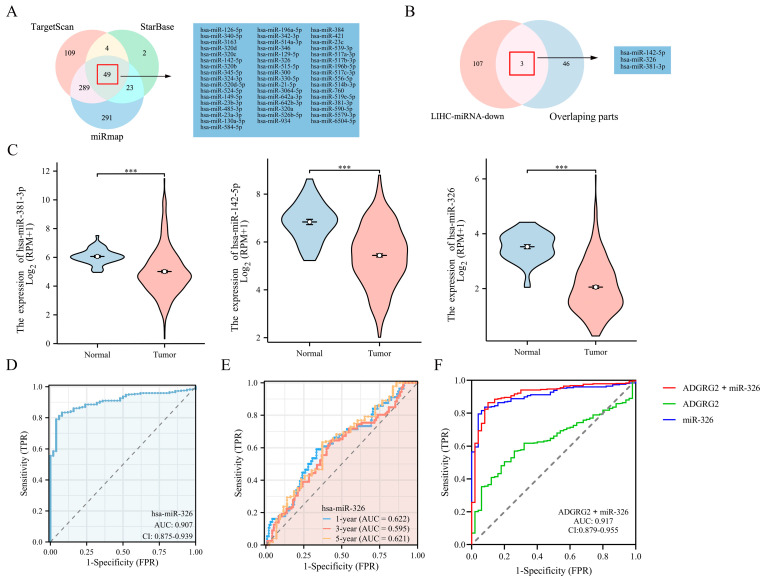
Prediction and diagnostic value of miRNAs targeting ADGRG2 in HCC. (**A**) Venn diagram showing the results for ADGRG2 targets, predicted using the TargetScan, StarBase, and miRmap databases. (**B**) Screening for miRNAs with low expression in HCC and targeting ADGRG2. (**C**) The expression of miR-381-3p, miR-142-5p, and miR-326 in HCC. (**D**) The ROC and (**E**) time-dependent curves of miR-326 in HCC. (**F**) ROC curves of the combination of ADGRG2 and miR-326 for HCC. *** *p* < 0.001.

Through TargetScan, StarBase, and miRmap database predictions, the following 49 miRNAs were jointly predicted (Figure 9A). Based on the classic reverse regulatory connection between miRNA and target genes, three down-regulated miRNAs were identified, miR-381-3p, miR-142-5p, and miR-326 (Figure 9B,C). Moreover, miR-326 displayed a remarkable prognostic value, while the other two did not (Figure S3A–C). In contrast, it has been reported that a low expression of miR-326 has a poor prognosis in HCC patients [29]. Consequently, we chose miR-326 for further analysis and confirmed a low-level expression of miR-326 in HCC-paired samples (Figure S3D). The AUC of miR-326 was 0.909 according to the ROC curves (Figure 9D), and the AUC values of miR-326 at 1, 3, and 5 years were 0.622, 0.595, and 0.621, respectively (Figure 9E). Notably, the combined diagnosis of miR-326 and ADGRG2 showed a higher diagnostic potential (AUC = 0.917) (Figure 9F).

### 2.9. MiR-326 Suppressed the Proliferation and Migration of Liver Cancer Cells and Directly Targeted ADGRG2

**Figure 10:**
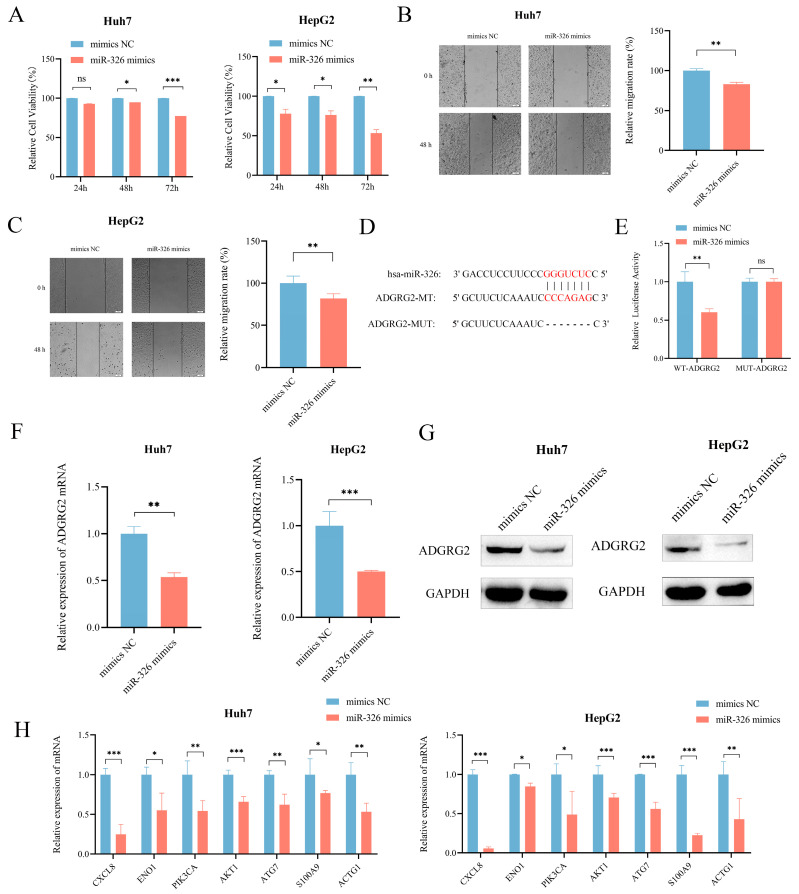
MiR-326 down-regulated the expression of ADGRG2 and suppressed the proliferation and migration of HCC cells. (**A**) The growth of Huh7 and HepG2 cells was measured using the CCK-8 assay after transfection of miR-326 mimics at 24 h, 48 h, and 72 h. (**B**,**C**) The migration abilities of Huh7 and HepG2 cells were assessed via wound healing assay after transfection of miR-326 mimics at 0 h and 48 h. (**D**) Hypothetical binding site of ADGRG2 and miR-326. (**E**) Interaction between ADGRG2 and miR-326, assessed via luciferase reporter assay. (**F**) Relative expression of ADGRG2 in Huh7 cell that were overexpressed with miR-326 by RT-qPCR. (**G**) Western blot analysis of the ADGRG2 protein level after transfection with NC and miR-326 mimics in Huh7 and HepG2 cells. (**H**) Relative mRNA expression of NET-related genes in Huh7 and HepG2 cells overexpressed with miR-326. * *p* < 0.05, ** *p* < 0.01, *** *p* < 0.001, ns: no significance.

We then further evaluated the probable regulatory role of miR-326 in HCC. CCK-8 assay and wound healing assays revealed that miR-326 mimics could effectively inhibit the growth and migration of Huh7 and HepG2 cells separately (Figure 10A–C). In addition, in order to further investigate whether miR-326 directly targets ADGRG2, we predicted their binding sites (Figure 10D) and found that the luciferase reporter gene containing a wild-type binding site was reduced sufficiently by miR-326 in HEK 293T cells (*p* < 0.01; Figure 10E). RT-qPCR and Western blot were applied individually to discover that the mRNA and protein levels of ADGRG2 were significantly down-regulated in Huh7 and HepG2 cells after overexpression of miR-326 (Figure 10F,G). In addition, we verified that the mRNA levels of NET-related genes were also down-regulated by miR-326 (Figure 10H).

## 3. Discussion

ADGRG2 is a member of the GPCRs family, many of which are already drug targets [23]. However, its role in HCC has rarely been investigated. In the present study, we observed that ADGRG2 was highly expressed in HCC tissues and HCC cell lines (Figure 1). A high expression of ADGRG2 was associated with poor clinical factors and poor prognosis in HCC patients (Table 1 and Figure 2A). Univariate and multivariate regression analysis confirmed that ADGRG2 expression was independently predictive of the clinical outcomes of HCC patients (Figure 2C,D). ROC curve analysis also illustrated that ADGRG2 could diagnose HCC (Figure 2B). AFP is a widely used biomarker for screening hepatocellular carcinoma, which had a sensitivity and specificity of only 39–64% and 76–91%, respectively [30]. For this reason, additional biomarkers need to be added. This study found a significant correlation between the expression of ADGRG2 and AFP (*p* < 0.01) in HCC (Table 1). Most notably, patients with AFP (≤400 ng/mL) and a high expression of ADGRG2 were more ill than those with other characteristics (Table 1), and had a worse OS than the ADGRG2 low-expression group (*p* < 0.05) (Figure S1). Among these patients, there were more immune checkpoints that were markedly and positively correlated with ADGRG2 (Figure S2C). Therefore, we speculated that ADGRG2 could provide a new monitoring pathway for HCC patients with AFP ≤ 400 ng/mL.

The GSEA analysis highlighted that ADGRG2-related genes were associated with hallmarks of cancer cells, such as adhesion, cytoskeleton, oxidative phosphorylation, and protein glycosylation (Figure 3B). During the epithelial mesenchymal transformation (EMT), the change in adhesion ability and the dramatic reorganization of the actin cytoskeleton make cancer cells have the characteristics of migration and invasion [31]. Most tumor cells rely on aerobic glycolysis rather than oxidative phosphorylation to generate their energy, known as the Warburg effect [32]. Malignant tumor transformation was associated with abnormal glycosylation, and there was evidence that O-GlcNAcylation was highly linked to the onset, growth, invasion, and metastasis of HCC [33]. Meanwhile, ADGRG2 was associated with cancer-related signaling pathways such as JAK-STAT, MAPK, calcium, and the PPAR signaling pathway (Figure 3B). Up to now, numerous studies have revealed that the JAK-STAT, MAPK, and calcium signaling pathways can induce proliferation, migration, and invasion, which are strongly connected to the occurrence and metastasis of liver cancer [17,34,35]. The expression of ADGRG2 was negatively correlated with the PPAR signaling pathway, and one of the members of PPARγ mainly mediated the antiangiogenic process and may be a therapeutic target for liver cancer [36]. Herein, we validated that silencing ADGRG2 can effectively inhibit the growth and migration of HepG2 and Huh7 cell lines (Figure 4). It was speculated that ADGRG2 may participate in the progression of HCC.

On the other hand, the functional enrichment analysis showed that ADGRG2 was considerably enriched in immune-related components and pathways, such as the cytokine–cytokine receptor interaction, chemokine signaling pathway, and IL-17 signaling pathway (Figure 3B–E). Subsequent immune infiltration assessment revealed that ADGRG2 was significantly correlated with macrophages, neutrophils, and Th2 cells, especially neutrophils, which was calculated using five algorithms (Figure 5C). As an essential component in TME, neutrophils and their activation have been proven to promote carcinogenesis [37]. Activated neutrophils release neutrophil extracellular traps (NETs), a chromatin- and granular-protein-based network structure, which possess a tumor-promoting activity of driving cancer growth, invasion, metastasis, and angiogenesis, and are associated with a poor prognosis [38]. Studies also found that NETs induced inflammation and enhanced metastasis in HCC [39]. Next, we screened a total of 16 NET-related genes in HCC and found that 11 of them have a strong positive correlation with ADGRG2 (Figure 5D). Further LASSO analysis revealed eight nonzero coefficients of NETs- and ADGRG2-related genes in liver cancer, all of which may serve as independent prognostic markers for HCC patients. Finally, we found that HCC patients with high NET scores had a poor prognosis (Figure 5H). In addition, we verified that the mRNA levels of seven the of NET-related genes were significantly down-regulated after silencing ADGRG2 (Figure 5I). It is worth noting that ENO1 has been identified to be overexpressed in over 70 percent of global cancers [40], covering highly metastatic HCC cells, and predicted a worse prognosis of HCC [41]. Studies have shown that HBV infection may enhance the expression of S100A9 and accelerate the production of NETs, and it is possible for serum S100A9 to distinguish the grade of liver necrosis and inflammation [42,43]. To summarize, ADGRG2 may be closely related to neutrophils and NETs and influence the tumor microenvironment to promote the formation and progression of HCC.

Sustained inflammation promotes the development of hepatic fibrosis into cirrhosis and, ultimately, hepatocellular carcinogenesis [44]. Chemokines and cytokines can recruit inflammatory cells and trigger chronic inflammatory diseases and play an important role in cancer [45]. We found a remarkable positive correlation between ADGRG2 and most chemokines and their receptors (Tables S3 and S4). It is worth noting that the CCL20-CCR6 axis, CCL1-CCR8 axis, and CCL28-CCR axis can all induce the proliferation and migration of liver cancer cells, recruit immunosuppressive cells TAM and Tregs, and promote immune tumor escape [46]. The high-expression group of ADGRG2 was associated with immune inflammatory cells, including macrophages, Th2 cells, and neutrophils (Figure 5B). Tumor-associated macrophages (TAMs) are an essential class of immune cells in TME, composed of M2 and a small fraction of M1 macrophages [47]. M1 macrophages typically have pro-inflammatory effects, while M2 macrophages have anti-inflammatory effects [48]. Both types of macrophages are required in HCC, the former being required for the initiation of liver cancer and the latter being required for the maintenance of the disease [49]. It has been reported that the increase in Th2 cells in HCC patients led to an increased likelihood of carcinogenesis in chronic HCV patients [50]. Additionally, the expression of ADGRG2 is substantially positively related to inflammatory cytokines, and data showed a marked increase in ADGRG2 in patients with liver cirrhosis and hepatocellular carcinoma from GSE89377 (Figure 6A,B). Altogether, AGDGR2 may be involved in the inflammatory process of liver cancer development.

Immunotherapy has been recommended as a potential treatment option for hepatocellular carcinoma in recent years, particularly targeting immune checkpoints [51]. Our findings suggested that a high ADGRG2 expression was associated with an increased expression of CD274, HAVCR2, VTCN1, and VEG41FA, and the ADGRG2 high-expression group has a higher IPS score and more responses after receiving anti-PD-1 therapy, predicting a better response to immunotherapy in ADGRG2 high-expression patients (Figure 7). Recently, the combination of some immune checkpoint inhibitors and some small molecule drugs has been a promising method for treating cancer. One clinical data showed that the combination of lenvatinib with an anti-PD-1 antibody (pembrolizumab) was highly effective [52]. Thereby, two drugs that were sensitive to the ADGRG2 high-expression group, namely, PIK-93 and NPK76-II-72-1, were screened and verified via molecular docking (Figure 8). As a PI4-kinase (PI4K) inhibitor, PIK-93 was found to reduce PD-L1 expression in tumor cells and M1 macrophages and suppress tumor growth when combined with an anti-PD-L1 antibody [53]. There are very few studies on NPK76-II-72-1: only one study mentioning that NPK76-II-72-1 regulated the cell cycle by targeting Polo-like kinase 3 (PLK3) [54]. The above findings could contribute to the development of candidate drugs for HCC and guide the prognosis of patients that are treated with immunotherapy.

Based on the biological significance of miRNAs for disease diagnosis, prognosis, and regulation of target genes, we predicted miRNAs targeting ADGRG2 via bioinformatics, wherein miR-326 was a miRNA with a striking negative correlation with ADGRG2 expression and diagnostic value (Figure 9). Although our analysis demonstrated that patients with a high miR-326 expression had an unfavorable prognosis (Figure S3C), another study showed a favorable prognosis for HCC patients with elevated miR-326 expression [29], which may be related to the different sources of tissue samples. Therefore, our in vitro experiments displayed that the increased expression of miR-326 markedly lowered the cell viability of Huh7 and HepG2 in HCC cells and inhibited cell migration (Figure 10A–C), again validating miR-326 as a tumor suppressor in HCC, which was consistent with the latest findings [29,55,56]. Further, we confirmed that miR-326 could directly target the 3’-UTR of ADGRG2 and down-regulate the mRNA and protein expression levels of ADGRG2 in HCC cells via dual luciferase reporter assay, qPCR, and Western blot (Figure 10D–G). Seven NET-related genes were down-regulated by miR-326 (Figure 10H), which was consistent with the trend after ADGRG2 silencing. In short, we considered miR-326 can serve as a marker for the diagnosis of HCC in combination with ADGRG2, and we discovered for the first time that miR-326 can target AGDGR2 to inhibit the proliferation and migration of the HCC cells Huh7 and HepG2, which are related to NETs.

This is the first exploration of the potential role of ADGRG2 in HCC. However, our study still has limitations. We mainly used a variety of bioinformatics methods to predict the potential biological effects of ADGRG2, and they need to be validated clinically through large cohorts and multicenter studies. Future studies could also verify the molecular mechanism of ADGRG2 related to NETs and drug actions through cell lines, animal models, and human samples and further analyze and study the interaction of lncRNA with miR-326 through the competitive endogenous RNA (ceRNA) network.

## 4. Materials and Methods

### 4.1. Data Download and Processing

Clinical data and ADGRG2 expression data of the TCGA and GEO cohort (GSE101685, GSE87630, GSE89377, and GSE91061) were retrieved from the Genomic Data Commons (GDC) database (https://portal.gdc.cancer.gov/, accessed on 12 April 2023), ICGC portal (https://dcc.icgc.org/, accessed on 13 April 2023), and GEO database (https://www.ncbi.nlm.nih.gov/geo/, accessed on 14 April 2023), respectively. The R package “Limma” was used to assess the difference in RNA levels of expression between the GSE1016851 and GSE87630 chips. The TCGA_LIHC included 374 HCC tumor tissues and 50 TCGA-paired normal tissues. Moreover, immunohistochemical pictures of ADGRG2 were derived from the Human Protein Atlas database (https://www.proteinatlas.org/, accessed on 15 April 2023), and the immunohistochemistry (IHC) score was evaluated using the IHC profiler [57]. The GSE91061 melanoma immunotherapy cohort was used to validate the ability of ADGRG2 immunotherapy by including 51 samples following anti-PD-1 treatment, where responders included complete response (CR) or partial response (PR), and nonresponders included progress disease (PD). Given the controversial role of stable disease (SD) in response to treatment, it was not included [58].

### 4.2. Identification of ADGRG2-Related Genes

HCC samples in the TCGA database were grouped into low- and high-ADGRG2 expression based on median expression values of ADGRG2, and differentially expressed genes (DEGs) between both groups were calculated using the “DESeq2” R package, with adjusted *p*-values < 0.05 and |log2 FC| ≥ 1 as cut-off values.

### 4.3. Enrichment Analysis of ADGRG2-Related DEGs in HCC

Gene set enrichment analysis (GSEA) with 1000 permutations was carried out to identify the function and pathways of ADGRG2-related genes (c2.cp.kegg.v7.2.symbols.gmt) [59]. It was considered statistically significant when the standardized enrichment score |(NES)| > 1.5 with the adjusted *p*-value < 0.05. In addition, 881 DEGs were uploaded to the STRING database for further screening to obtain 84 hub DEGs (high confidence > 0.7, species “Homo sapiens”, hidden free nodes), and Cytoscape 3.9.1 software was utilized for illustrating the protein interaction network. Gene Ontology (GO) and Kyoto Encyclopedia of Genes and Genomes (KEGG) enrichment analyses of the hub DEGs were accomplished by means of the R package “clusterProfiler”.

### 4.4. Identification of Potential miRNAs Targeting ADGRG2

The target miRNAs of ADGRG2 were predicted and analyzed using StarBase (https://starbase.sysu.edu.cn/, accessed on 24 April 2023), miRmap databases (https://mirmap.ezlab.org/, accessed on 24 April 2023), and TargetScan (https://www.targetscan.org/, accessed on 24 April 2023) and screened via negative correlation with ADGRG2 expression.

### 4.5. Correlation Analysis of ADGRG2 and Immunity Characteristics

Single sample gene set enrichment algorithm (ssGSEA) based on R package “GSVA” was executed to calculate the immune infiltration levels of 24 immune cells in HCC samples [60], and the Wilcoxon signed-rank test was applied to compare the difference of tumor-infiltrating immune cells between high- and low-ADGRG2 groups. TIMER2 database (http://timer.cistrome.org/, accessed on 26 April 2023) was chosen to measure the correlation between ADGRG2 and neutrophils. Eighty-seven genes from previous studies were applied as initial biomarkers of neutrophil extracellular traps (NETs) [61,62]. To screen for core NET-related genes in association with HCC prognosis, univariate Cox analysis and LASSO algorithm were performed and a Cox proportional hazard regression model was constructed, which evaluated the association between the expression of each gene characterized by NETs in the TCGA_LIHC cohort and the overall survival (OS) of patients. Based on this model, if the regression coefficient for each NET-related gene was β, the NET score for each patient was as follows:

$$\mathrm{NETs}\;\mathrm{score}\;=\overset{n}{\underset{i=1}{\sum }}G_{i}\;\beta_{i}$$

where *n* was the number of NET signature genes, *Gi* was the gene *i*’s normalized expression level, *βi* was Cox’s regression coefficient for gene *i*, and the median NET score was considered to be the threshold dividing the tumor samples into high and low NET score groups. Finally, the prognostic value of the NET score was assessed. To illustrate the correlation between ADGRG2 and inflammatory cytokines, the R packages “ggplot2” and “pheatmap” were performed.

### 4.6. Prediction of Response to Immunotherapy

Correlative studies on ADGRG2 and 22 inhibitory immune checkpoint molecules were analyzed [63], and the TCIA database (https://www.tcia.at/home, accessed on 26 April 2023) was used to predict the clinical response to immunotherapy between low- and high-expressing ADGRG2 groups. Immunophenoscore (IPS) was used as a measure of tumor immunogenicity in the range of 0–10. The higher the IPS score, the stronger the immunogenicity.

### 4.7. Drug Sensitivity Analysis and Molecular Docking

In the R environment, the IC50 (half of inhibitory concentration) of some antitumor medicines in both groups was processed using the “pRRophetic” package. The “ggplot2” R package was used to generate box plots and scatter plots. Finally, Autodock Vina was used to perform molecular docking on the selected drugs [64], and a model of the drug and ADGRG2 protein was established using molecular docking. The higher the affinity, the lower the binding energy, and the binding energy is less than −5 kcal/mol, indicating that the drug is most likely to bind to the target protein, and that the affinity is good.

### 4.8. Cell Culture and Transfection

Huh7, HepG2, LO2, and HEK 293T cells were kindly provided by the Beijing Institute of Basic Medical Sciences and maintained in DMEM (Gibco, Waltham, MA, USA) containing 10% fetal bovine serum (FBS) at 37 °C, using a 5% CO_2_ incubator. ADGRG2 siRNA and its corresponding control (si-NC) were synthesized by GenePharma (Shanghai, China), and the following were the sequences: si-ADGRG2: 5′-CAUUACGGUGGUGGGAUAUTT-3′. MiR-326 mimics and negative control (NC) were produced by RiboBio (Guangzhou, China). RNA and plasmid transfection were performed according to the instructions of siRNA-mate and GP-transfect-Mate (GenePharma, Shanghai, China), respectively.

### 4.9. Reverse Transcription and Quantitative PCR (RT-qPCR)

TRIzol reagent (Thermo Fisher, Walsam, MA, USA) was used to isolate total mRNA at 48 h after transfection. RT-qPCR was conducted with the PerfectStart Uni RT and qPCR kits (Transgenic, Beijing, China). According to the 2^−ΔΔCT^ method, GAPDH was chosen as the internal reference gene to calculate the ratio of expression. The primer sequences used for RT-qPCR were described in Table S4.

### 4.10. CCK8 and Wound Healing Assays

The proliferation capacity was measured using the Cell Counting Kit-8 (Beyotime, Shanghai, China). HCC cells (5000 cells/well) were seeded into 96-well plates. At 24 h, 48 h, and 72 h after transfection, 100 μL of fresh medium containing 10% CCK-8 reagent was added to each of the wells. After an incubation period of 2 h at 37 °C, the absorbance was determined with a microtiter plate reader (PerkinElmer, Waltham, MA, USA) at 450 nm. The migration ability was estimated via the wound healing assay. In 12-well plates, transfected HCC cells were placed, and when the cells were allowed to reach 100% confluence, the sterile pipet tips were used to scratch the bottom of the culture plate. The cells were gently rinsed with serum-free medium 2–3 times and then cultivated with serum-free medium. Under an inverted microscope (Olympus, Tokyo, Japan), the cells were photographed after 0 h and 48 h, respectively.

### 4.11. Dual Luciferase Reporter Assay

The binding site of miR-326 to ADGRG2 3’-UTR was predicted using StarBase database, and the binding fragment of miR-326 to ADGRG2 3’-UTR was amplified via PCR. To generate the wild-type (WT) ADGRG2 plasmid, the amplified product was introduced into the PGL3 promoter plasmid vector. ADGRG2 mutant (MUT) plasmid was constructed via gene mutation technique. HEK-293 T cells were co-transfected with miR-326 mimics (or mimics NC) and plasmids. After 48 h of transfection, the cells were collected, and the fluorescence value was determined via a dual luciferase reporter gene detection system (Transgenic, Beijing, China).

### 4.12. Western Blot Analysis

The transfected HCC cells were lysed with RIPA buffer (Beyotime, Shanghai, China), and the concentration of protein was determined via enhanced BCA method (Beyotime, Shanghai, China). Samples were separated using an 8% SDS-PAGE gel and then transferred to PVDF membranes (Millipore, Burlington, MA, USA). Then, 5% skim milk was used to block at room temperature for 1 h. The primary antibodies ADGRG2 (1:1000, Proteintech, Chicago, IL, USA) and GAPDH (1:20,000, Proteintech, Chicago, IL, USA) were incubated overnight at 4 °C, and the second antibody (1:3000, Beyotime, Shanghai, China) was added the next day. BeyoECL Plus Kit (Beyotime, Shanghai, China) and ChemiDoc XRS imaging system (Bio-Rad, Hercules, CA, USA) were utilized to visualize the bands.

### 4.13. Statistical Analysis

GraphPad Prism 8.1 software for Student’s *t*-test was used for cell experiments. Kaplan–Meier, Cox regression, and receiver operating characteristic (ROC) curve analysis were performed using the “survival”, “pROC”, and R package. Statistical data analysis was completed using R software (version 4.2.1) and GraphPad Prism 8.1. *p* < 0.05 was regarded as statistically noteworthy.

## 5. Conclusions

In summary, our findings suggested that ADGRG2 expression was highly overexpressed in HCC and was associated with an unfavorable prognosis, especially when AFP ≤ 400 ng/mL. ADGRG2 not only enhanced the proliferation and migration of liver cancer cells but was also closely related to tumor immune infiltration and immune checkpoints. ADGRG2 may affect tumor immunity and the inflammatory microenvironment through collecting neutrophils and further releasing NETs. The high expression of ADGRG2 indicated a better immune therapy effect. Therefore, ADGRG2 may serve as a promising biomarker and potential therapeutic target for the diagnosis, immunotherapy, and prognosis evaluation of HCC. In addition, research has found that miR-326 inhibited the proliferation and migration of hepatocellular carcinoma cells by targeting ADGRG2, and the combination of ADGRG2 and miR-326 exhibited better diagnostic potential. The two screened anticancer drugs PIK-93 and NPK76-II-72-1 may target ADGRG2, providing new strategies for diagnosing and treating HCC.

## Figures and Tables

**Table 1:** The correlation between ADGRG2 expression level and clinicopathological factors in HCC.

Characteristics	Low Expression of ADGRG2	High Expression of ADGRG2	*p* Value
Pathologic stage, *n* (%)			0.03174037
Stage I	96 (36.9%)	77 (29.6%)	
Stage II	36 (13.8%)	51 (19.6%)	
AFP (ng/mL), *n* (%)			0.00295382
≤400	97 (34.6%)	118 (42.1%)	
>400	43 (15.4%)	22 (7.9%)	
OS event, *n* (%)			0.02987853
Alive	132 (35.3%)	112 (29.9%)	
Dead	55 (14.7%)	75 (20.1%)	

**Table 2:** Association of *ADGRG2* expression with neutrophil markers in HCC.

Marker	Marker Type	Correlation	*p* Value	Marker	Marker Type	Correlation	*p* Value
CD15	TAN	0.374	9.95 × 10^−14^	CD54	TAN	0.243	2.08 × 10^−6^
CXCR1	inflammation	0.126	1.51 × 10^−2^	CD217	inflammation	0.165	1.44 × 10^−3^
CXCR2	TAN, inflammation	0.219	2.02 × 10^−5^	ITGB2	inflammation	0.147	4.68 × 10^−3^
CD11b	TAN, inflammation	0.265	2.33 × 10^−7^	PTPRC	TAN	0.154	2.99 × 10^−3^
CD11c	inflammation	0.143	5.90 × 10^−3^	CD43	inflammation	0.113	2.95 × 10^−2^
FCGR3A	TAN, inflammation	0.189	2.55 × 10^−4^	TLR2	inflammation	0.253	7.67 × 10^−7^
HLA-DRA	TAN	0.228	9.39 × 10^−6^	TLR4	inflammation	0.26	3.64 × 10^−7^
HLA-DRB1	TAN	0.143	5.91 × 10^−3^	TLR5	inflammation	0.348	4.96 × 10^−12^
HLA-DRB3	TAN	0.181	4.43 × 10^−4^	TLR7	inflammation	0.243	2.15 × 10^−6^
CD49d	inflammation	0.169	1.05 × 10^−3^	TLR8	inflammation	0.14	7.07 × 10^−3^
ARG1	TAN	0.108	3.79 × 10^−2^	TLR9	inflammation	0.165	1.42 × 10^−3^
CD86	TAN	0.147	4.65 × 10^−3^	CD63	inflammation	0.144	5.58 × 10^−3^

## Data Availability

Data are contained within the article and supplementary materials.

## Supplementary Materials

The following supporting information can be downloaded at https://www.mdpi.com/article/10.3390/ijms242316986/s1.
