# Supplementary material for: Adhesion G Protein-Coupled Receptor G2 Promotes Hepatocellular Carcinoma Progression and Serves as a Neutrophil-Related Prognostic Biomarker
Source: Int J Mol Sci. 2023 Nov 30;24(23):16986. doi: 10.3390/ijms242316986 (PMC10707058; doi:10.3390/ijms242316986)
Supplement: Supplementary file 1 [file ijms-24-16986-s001.zip › ijms-2684306-supplementary.pdf]

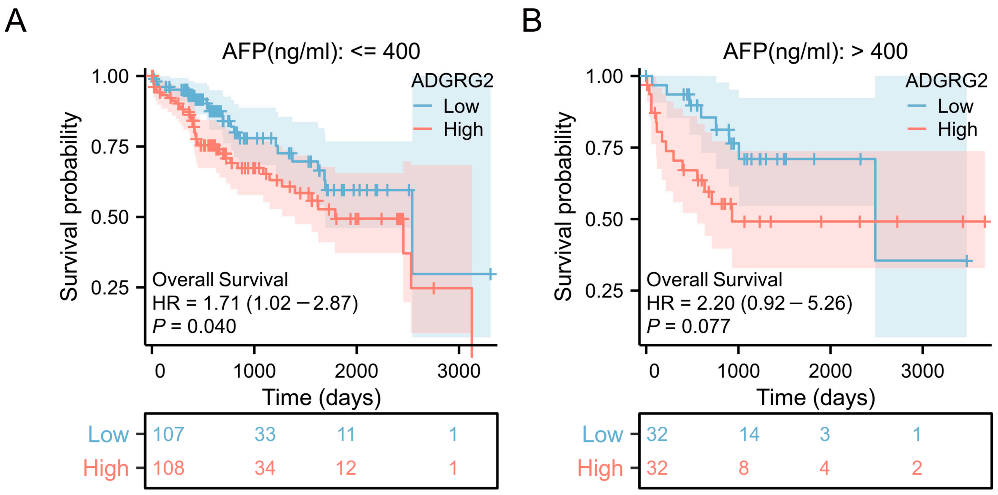

Figure S1. The clinical subgroup survival curves for patients with HCC presenting with high and low ADGRG2 expression. (A) AFP ≤ 400 ng/ml. (B) AFP > 400 ng/ml.

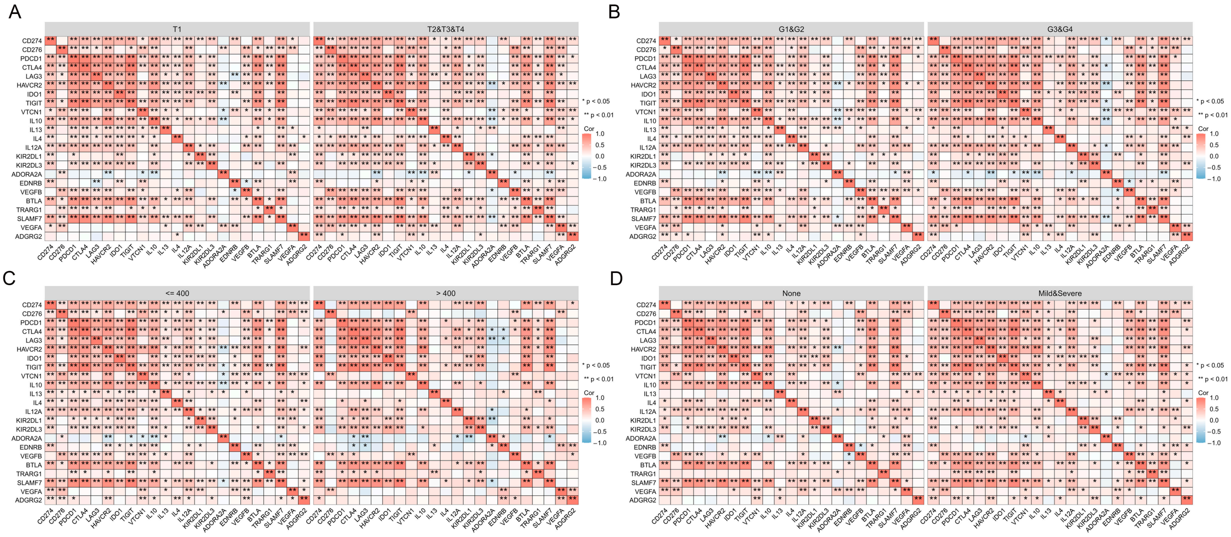

Figure S2. The correlation between immune checkpoints and *ADGRG2* in clinical subgroups. (A） Pathologic_T_stage (T1 vs T2&T3&T4). (B) Histologic_grade (G1&G2 vs G3&G4). (C) AFP (≤ 400 ng/ml vs > 400 ng/ml). (D) Adjacent_hepatic_tissue_inflammation (None vs Mild&Severe).

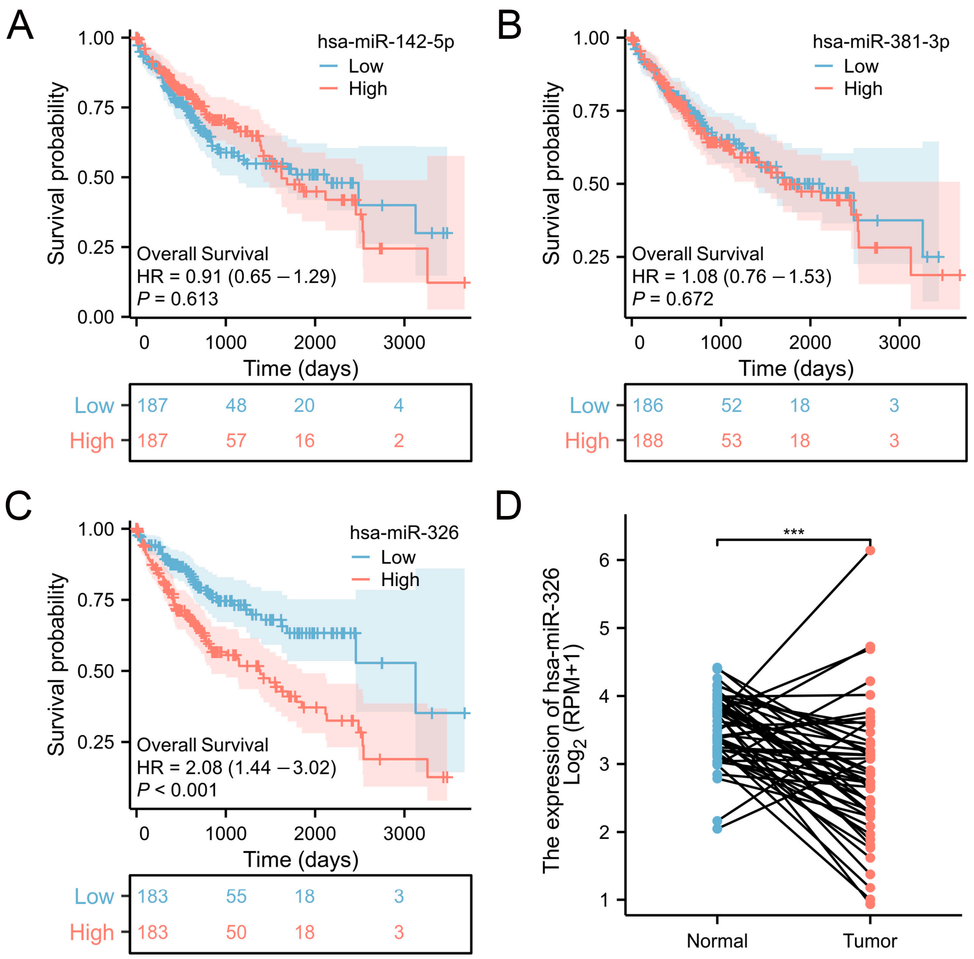

Figure S3. The prognostic value of miRNA expression in patients with HCC. (A-C) miR-142-5p, miR-381-3p, miR-326. (D) The expression of miR-326 in TCGA paired samples.

Table S1. Sixteen NETs-related genes with prognostic potential.

Gene_name	Gene_id	HR	CI	p.Cox
AKT1	ENSG00000142208	1.811	1.272-2.579	0.000992
ATG7	ENSG00000197548	1.768	1.243-2.515	0.001539
S100A9	ENSG00000163220	1.522	1.073-2.157	0.018395
ENO1	ENSG00000074800	1.982	1.390-2.826	0.000157
ACTN4	ENSG00000130402	1.419	1.004-2.006	0.04752
ACTN1	ENSG00000072110	1.444	1.022-2.041	0.037306
ACTG1	ENSG00000184009	1.568	1.106-2.221	0.01143
ACTB	ENSG00000075624	1.472	1.038-2.087	0.030127
TK1	ENSG00000167900	1.457	1.030-2.061	0.033187
MNDA	ENSG00000163563	1.570	1.101-2.239	0.012645
SELP	ENSG00000174175	0.687	0.485-0.973	0.034431
PIK3CA	ENSG00000121879	1.458	1.026-2.072	0.035419
CYBB	ENSG00000165168	1.474	1.037-2.095	0.030489
CXCL8	ENSG00000169429	1.737	1.217-2.477	0.002327
MAPK3	ENSG00000102882	1.777	1.252-2.523	0.001298
MMP9	ENSG00000100985	1.856	1.303-2.643	0.000616

Table S2. Correlation between ADGRG2 expression and chemokines in HCC.

Chemokine	Correlation	*p* Value	Chemokine	Correlation	*p* Value
CCL1	0.109	3.55×10^{-2}	CCL26	0.248	1.31×10^{-6}
CCL2	0.137	8.07×10^{-3}	CCL27	-0.005	9.21×10^{-1}
CCL3	0.01	8.41×10^{-1}	CCL28	0.194	1.69×10^{-4}
CCL4	0.062	2.36×10^{-1}	CX3CL1	-0.055	2.93×10^{-1}
CCL5	0.098	5.84×10^{-2}	CXCL1	0.305	1.99×10^{-9}
CCL7	0.062	2.31×10^{-1}	CXCL2	0.122	1.9×10^{-2}
CCL8	0.044	3.97×10^{-1}	CXCL3	0.235	4.87×10^{-6}
CCL11	0.194	1.71×10^{-4}	CXCL5	0.212	3.99×10^{-5}
CCL13	0.1	5.38×10^{-2}	CXCL6	0.275	7.53×10^{-8}
CCL14	-0.071	1.74×10^{-1}	CXCL8	0.305	1.98×10^{-9}
CCL15	0.026	6.22×10^{-1}	CXCL9	0.106	4.11×10^{-2}
CCL16	-0.191	2.08×10^{-4}	CXCL10	0.152	3.26×10^{-3}
CCL17	0.073	1.61×10^{-1}	CXCL11	0.214	3.19×10^{-5}
CCL18	-0.029	5.74×10^{-1}	CXCL12	0.005	9.31×10^{-1}
CCL19	0.099	5.61×10^{-2}	CXCL13	0.223	1.49×10^{-5}
CCL20	0.141	6.56×10^{-3}	CXCL14	0.118	2.25×10^{-2}
CCL21	0.073	1.6×10^{-1}	CXCL16	0.177	6.17×10^{-4}
CCL22	0.11	3.37×10^{-2}	CXCL17	-0.04	4.43×10^{-1}
CCL23	0.026	6.17×10^{-1}	XCL1	0.026	6.24×10^{-1}
CCL24	-0.013	8.06×10^{-1}	XCL2	0.008	9.02×10^{-2}
CCL25	0.094	6.91×10^{-2}			

Table S3. Correlation between *ADGRG2* expression and chemokine receptors in HCC.

Chemokine receptor	Correlation	*p* Value
CCR1	0.17	9.84×10^{-4}
CCR2	0.174	7.51×10^{-4}
CCR3	0.154	5.13×10^{-3}
CCR4	0.151	3.55×10^{-3}
CCR5	0.138	7.78×10^{-3}
CCR6	0.114	2.87×10^{-2}
CCR7	0.051	3.23×10^{-1}
CCR8	0.156	2.67×10^{-3}
CCR9	0.164	1.55×10^{-3}
CCR10	0.094	7.1×10^{-2}
CX3CR1	0.174	7.4×10^{-4}
CXCR1	0.126	1.51×10^{-2}
CXCR2	0.219	2.02×10^{-5}
CXCR3	0.13	1.24×10^{-2}
CXCR4	0.116	2.53×10^{-2}
CXCR5	0.051	3.26×10^{-1}
CXCR6	0.15	3.79×10^{-3}
XCR1	0.135	9.4×10^{-3}

Table S4. Primers for quantitative RT-PCR.

Gene	Forward 5'-3'	Reverse 5'-3'
ADGRG2	ACGAATGGCACCTTAACTGG	ACAGCAGCAGTGTTCCATT
CXCL8	GCAGAGGGTTGTGGAGAAGT	AAGGCAGATACCTAATGACGATT
ENO1	CGAGACCCAGTGGCTAGAAG	ATGGGCTGTGGGTTCTAAGG
PIK3CA	TGGGACCCGATGCGGTTAG	TCACCTGATGATGGTCGTGG
AKT1	CAGCCTGGGTCAAAGAAGTC	CTCACGTTGGTCCACATCCT
ATG7	CTGTGGTTGCCGGAAGTTG	GTCCTTGGGAGCTTCATCC
S100A9	CCTCCCACGAGAAGATGCAC	CCTGGCCTCCTGATTAGTGG
ACTG1	CCGAGCCGTGTTTCCTTCC	GCCATGCTCAATGGGGTACT
GAPDH	CGACCACTTTGTCAAGCTCA	AGGGGTCTACATGGCAACTG
